# Dairy Product Consumption and Risk of Non-Hodgkin Lymphoma: A Meta-Analysis

**DOI:** 10.3390/nu8030120

**Published:** 2016-02-27

**Authors:** Jia Wang, Xutong Li, Dongfeng Zhang

**Affiliations:** 1 Department of Epidemiology and Health Statistics, the Medical College of Qingdao University, No. 38 Dengzhou Road, Qingdao 266021, China; wangjia900929@163.com; 2 Department of oncology, Second affiliated hospital, the Medical College of Qingdao University, Qingdao 266071, China; lixutong1@sina.com

**Keywords:** dairy product, milk, non-Hodgkin lymphoma, meta-analysis

## Abstract

Many epidemiologic studies have explored the association between dairy product consumption and the risk of non-Hodgkin lymphoma (NHL), but the results remain controversial. A literature search was performed in PubMed, Web of Science and Embase for relevant articles published up to October 2015. Pooled relative risks (RRs) with 95% confidence intervals (CIs) were calculated with a random-effects model. The dose-response relationship was assessed by restricted cubic spline. A total of 16 articles were eligible for this meta-analysis. The pooled RRs (95% CIs) of NHL for the highest *vs.* lowest category of the consumption of total dairy product, milk, butter, cheese, ice cream and yogurt were 1.20 (1.02, 1.42), 1.41 (1.08, 1.84), 1.31 (1.04, 1.65), 1.14 (0.96, 1.34), 1.57 (1.11, 2.20) and 0.78 (0.54, 1.12), respectively. In subgroup analyses, the positive association between total dairy product consumption and the risk of NHL was found among case-control studies (RR = 1.41, 95% CI: 1.17–1.70) but not among cohort studies (RR = 1.02, 95% CI: 0.88–1.17). The pooled RRs (95% CIs) of NHL were 1.21 (1.01, 1.46) for milk consumption in studies conducted in North America, and 1.24 (1.09, 1.40) for cheese consumption in studies that adopted validated food frequency questionnaires. In further analysis of NHL subtypes, we found statistically significant associations between the consumption of total dairy product (RR = 1.73, 95% CI: 1.22–2.45) and milk (RR = 1.49, 95% CI: 1.08–2.06) and the risk of diffuse large B-cell lymphoma. The dose-response analysis suggested that the risk of NHL increased by 5% (1.05 (1.00–1.10)) and 6% (1.06 (0.99–1.13)) for each 200 g/day increment of total dairy product and milk consumption, respectively. This meta-analysis suggested that dairy product consumption, but not yogurt, may increase the risk of NHL. More prospective cohort studies that investigate specific types of dairy product consumption are needed to confirm this conclusion.

## 1. Introduction

Dairy product is an important part of diet in many countries around the world. Itcontains many essential nutrients, such as fats, proteins, minerals, vitamin D, and other bioactive nutrients. Dairy product can increase the content of total body and lumbar spine bone mineral in children [1] and decrease the risk of cardiovascular disease [2], type 2 diabetes [3] and colorectal cancer [4]. However, in recent years, some studies have shown that excessive consumption of dairy product may be associated with several adverse health effects, for instance Parkinson’s disease [5] and prostate cancer [6]. Therefore, it is necessary to further explore the health effect of dairy product on non-Hodgkin lymphoma (NHL).

NHL refers to a heterogeneous group of malignant tumors of lymphoid tissue that differs from Hodgkin disease. Over the past few decades, the incidence and mortality of NHL has been increasing internationally [7,8,9]. It is estimated that there will be 71,850 new cases and 19,790 new deaths in the United States in 2015 [10]. In spite of the considerable public health significance, the etiology of NHL remains poorly understood. Recently, some studies have found that dietary factors may play a role in the development of NHL [11,12]. To date, several epidemiologic studies have explored the association between dairy product consumption and the risk of NHL. However, the results are inconsistent [13,14,15,16,17,18,19,20,21,22,23,24,25,26,27,28]. Therefore, we systematically conducted a meta-analysis to: (1) further explore the effect of total dairy product consumption on the risk of NHL; (2) further investigate the associations between specific types of dairy product consumption and the risk of NHL, including milk, butter, cheese, yogurt and ice cream; and (3) evaluate the possible dose-response relationships between the consumption of total dairy product and milk and the risk of NHL, respectively.

## 2. Materials and Methods

### 2.1. Literature Search Strategy

We conducted a literature search to identify relevant available articles published in English from PubMed, Web of Science and Embase up to October 2015. Search terms included “dairy” (or “milk” or “butter” or “cheese” or “yogurt” or “ice cream”) and “non-Hodgkin lymphoma” (or “non-Hodgkin’s lymphoma” or “NHL”). We also reviewed the reference lists of the included studies for undetected relevant studies.

### 2.2. Inclusion Criteria

The inclusion criteria are as follows: (1) case-control or cohort study published as an original study; (2) the exposure of interest were total dairy product, milk, butter, cheese, yogurt or ice cream; (3) the outcome of interest was non-Hodgkin lymphoma; (4) relative risk (RR) with 95% confidence interval (CI) (or data to calculate these) were provided; (5) the most recent and complete study was selected if data from the same population had been published more than once.

Two investigators (Jia Wang and Xutong Li) searched and reviewed all identified studies independently. If the two investigators disagreed about the eligibility of an article, it was resolved by consensus with a third reviewer (Dongfeng Zhang).

### 2.3. Data Extraction

The following information was extracted from each study by two investigators independently: first author’s name, publication year, country in which the study was conducted, study design, follow-up duration, age range or mean age at baseline, sample size and number of cases, dietary assessment method, the type of dairy product, RR (we presented all results as RR for simplicity) with 95% CI for the highest *versus* lowest category of the consumption of total dairy product and specific types of dairy product, and variables adjusted for in each studies.

For dose-response analysis, the number of cases and participants (person-years), and RR (95% CI) for each category of total dairy product and milk were extracted. The median or mean level of total dairy product and milk for each category was assigned to the corresponding RR for every study. If the upper boundary of the highest category was not provided, we supposed that the boundary had the same amplitude as the contiguous category. If intakes were reported in densities (*i.e.*, g/1000 kcal), we estimated the absolute intakes by the mean energy intake of the participants [21]. When studies reported intakes in servings or times per day/week/month and did not provide a serving size, we converted them into grams per day by standard units of 244 g for milk and 177 g for total dairy product on the basis of serving sizes reported in the United States Department of Agriculture National Nutrient Database for Standard Reference [6,29]. We extracted RRs adjusted for the most confounders in the original studies.

### 2.4. Statistical Analysis

Pooled measure was calculated as the inverse variance-weighted mean of the logarithm of RR with 95% CI to assess the strength of associations between the consumption of total dairy product and specific types of dairy product and the risk of NHL, respectively. The DerSimonian and Laird random effect model (REM) was used to combine study-specific RRs (95% CIs) [30]. The *I*^2^ was adopted to assess the heterogeneity between studies (*I*^2^ values of 0%, 25%, 50% and 75% represent no, low, moderate and high heterogeneity, respectively) [31]. Meta-regression with restricted maximum likelihood estimation was performed to explore the potentially important covariates that might exert substantial impacts on between-study heterogeneity. *P*-values from meta-regression were calculated with a permutation test of 1000 to control the spurious findings [32]. Subgroup analyses were performed by study design, continent where the studies were conducted and dietary assessment method. Influence analysis was performed with one study removed at a time to assess whether the results could have been affected markedly by a single study [33]. Small-study effect was assessed with visual inspection of the funnel plot and Egger’s test [34].

For dose-response analysis, a two-stage, random-effects, dose-response meta-analysis [35] was performed to compute the trend from the correlated log RR estimates across levels of total dairy product and milk, respectively. In the first stage, a restricted cubic spline model with three knots at the 10th, 50th, and 90th percentiles [36] of the levels of total dairy product and milk was estimated using generalized least-square regression, taking into account the correlation within each set of published RRs [37]. Then the study-specific estimates were combined using the restricted maximum likelihood method in a multivariate random-effects meta-analysis [38]. A *p*-value for nonlinearity was calculated by testing the null hypothesis that the coefficient of the second spline is equal to 0.

All statistical analyses were performed with STATA version 12.0 (Stata Corporation, College Station, TX, United States). All reported probabilities (*p*-values) were two-sided with *p* < 0.05 considered statistically significant.

## 3. Results

### 3.1. Literature Search and Study Characteristics

**Figure 1:**
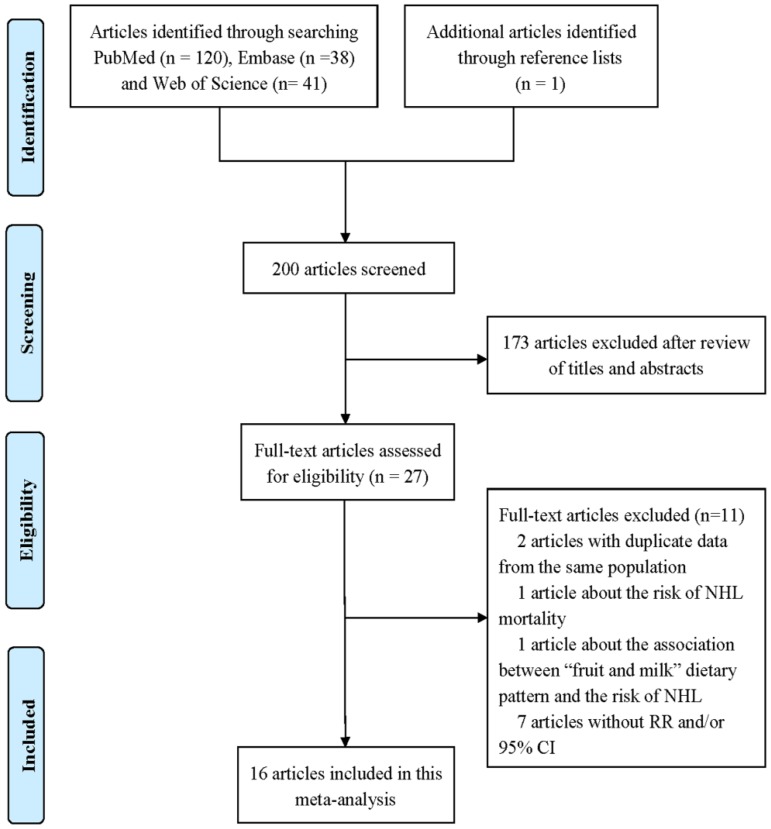
Flowchart of the selection of studies included in the meta-analysis.

We identified 199 articles by literature search, 173 of which were excluded after review of titles and abstracts (Figure 1). One additional article was found through the reference lists of included articles. Two articles with duplicate data from the same population, one article on the association between dairy product consumption and the risk of NHL mortality, one article on the association between “fruit and milk” dietary pattern and the risk of NHL, and seven articles without RR and/or 95% CI were excluded. Finally, 16 published articles [13,14,15,16,17,18,19,20,21,22,23,24,25,26,27,28] were eligible for this meta-analysis.

In these included articles, seven studies were conducted in North America, two in Latin America, four in Europe and three in Asia. Thirteen articles adopted validated food frequency questionnaires (FFQs) to assess the dietary consumption, and others used FFQs. With regard to study design, 13 articles were case-control studies, and three were cohort studies. The detailed characteristics of the included studies are shown in Table 1 and Table 2.

### 3.2. Quantitative Synthesis

The main results are summarized in Table 3.

#### 3.2.1. Total Dairy Product Consumption and the Risk of NHL

**Figure 2:**
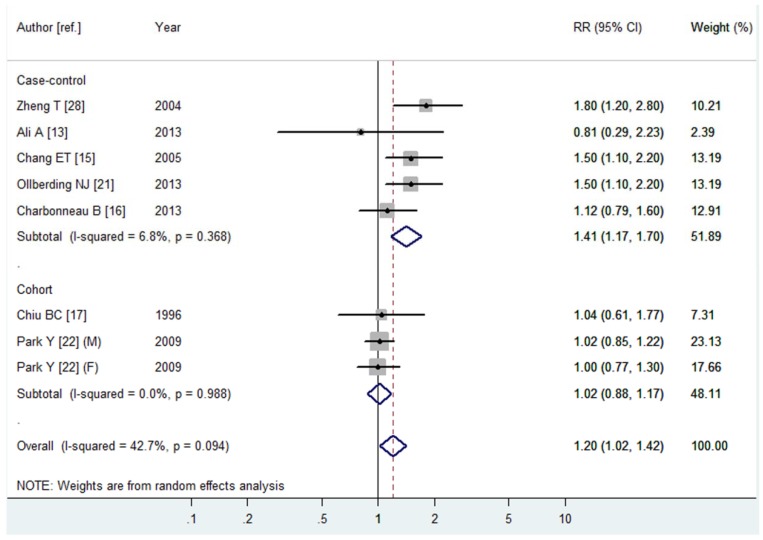
Forest plot of total dairy product consumption and the risk of NHL. The size of gray box is positively proportional to the weight assigned to each study, and horizontal lines represent the 95% confidence intervals.

Seven articles [13,15,16,17,21,22,28] with eight studies (five case-control studies and three cohort studies) were included, involving 4207 NHL cases. Among these studies, six were conducted in North America, one in Asia and one in Europe. All the studies adopted validated FFQs to assess total dairy product consumption. For the highest *vs.* lowest category of total dairy product consumption, the pooled RR of NHL was 1.20 (95% CI 1.02–1.42, *I*^2^ = 42.7%, *p*_heterogeneity_ = 0.094, Figure 2). In subgroup analysis stratified by study design, the pooled RRs in case-control and cohort studies were 1.41 (95% CI 1.17–1.70, *I*^2^ = 6.8%, *p*_heterogeneity_ = 0.368) and 1.02 (95% CI 0.88–1.17, *I*^2^ = 0.0%, *p*_heterogeneity_ = 0.988), respectively (Figure 2). In subgroup analysis stratified by continent in which the studies were conducted, the association between total dairy product consumption and the risk of NHL was not statistically significant among studies conducted in North America. In the further analysis of NHL subtypes, we just found a statistically significant association between total dairy product consumption and the risk of diffuse large B-cell lymphoma (DLBCL) (RR = 1.73, 95% CI 1.22–2.45, *I*^2^ = 0.0%, *p*_heterogeneity_ = 0.670).

**Figure 3:**
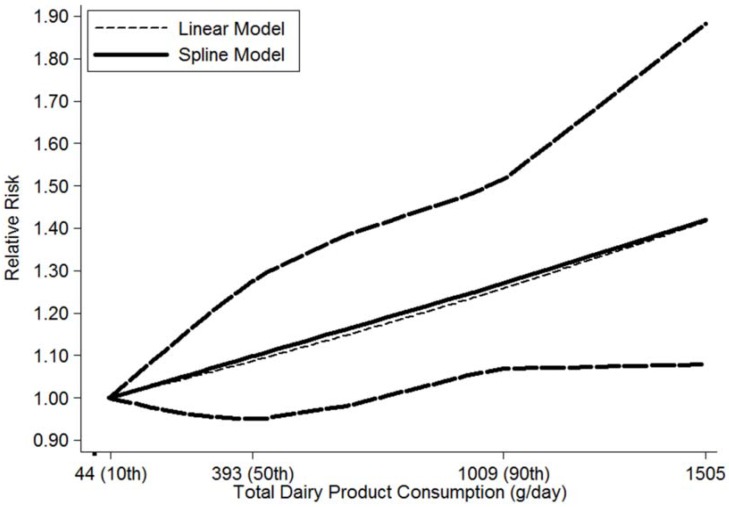
The dose-response analysis between total dairy product consumption and the risk of NHL with restricted cubic splines in a multivariate random-effects dose-response model. The solid line and the long dash line represent the estimated relative risks and their 95% CIs. Short dash line represents the linear relationship. The 10th, 50th and 90th percentiles represent three knots of total dairy product consumption.

For the dose-response analysis, data from five studies [13,15,16,17,21] were used, including 1679 NHL cases. A linear relationship was found between total dairy product consumption and the risk of NHL (*p*_nonlinearity_ = 0.91), and the RRs (95% CIs) of NHL were 1.03 (0.98–1.08), 1.10 (0.95–1.27), 1.19 (1.00–1.41), 1.27 (1.06–1.51) and 1.42 (1.06–1.89) for 150, 390, 730, 1000 and 1500 g/day compared with 40 g/day, respectively. In addition, the dose-response analysis suggested that NHL risk increased by 5% (1.05 (1.00–1.10)) for each 200 g/day increment of total dairy product consumption (Figure 3).

#### 3.2.2. Milk Consumption and the Risk of NHL

**Figure 4:**
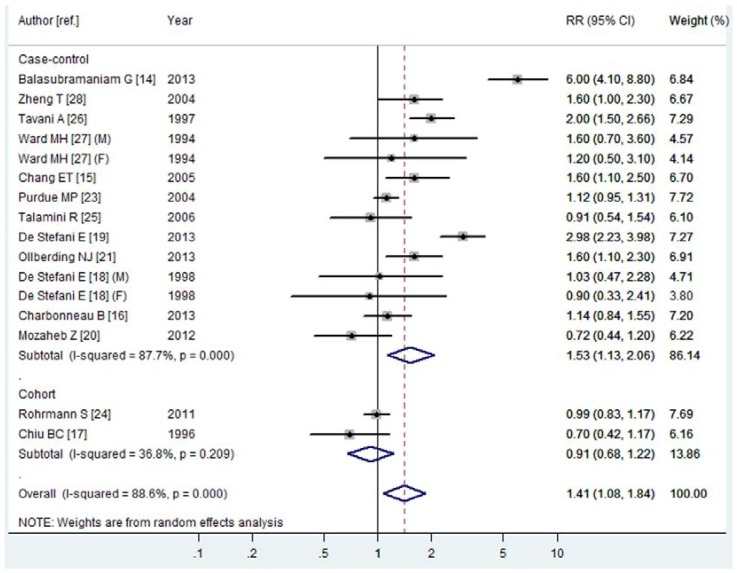
Forest plot of milk consumption and the risk of NHL. The size of the gray box is positively proportional to the weight assigned to each study, and horizontal lines represent the 95% confidence intervals.

Fourteen articles [14,15,16,17,18,19,20,21,23,24,25,26,27,28] with 16 studies (14 case-control studies and two cohort studies) were included, involving 7109 NHL cases. Among these studies, seven were conducted in North America, three in Latin America, four in Europe and two in Asia. Eleven studies adopted validated FFQs to assess milk consumption and five studies adopted FFQs. For the highest *vs.* lowest category of milk consumption, the pooled RR of NHL was 1.41 (95% CI 1.08–1.84, *I*^2^ = 88.6%, *P*_heterogeneity_ = 0.000, Figure 4). In subgroup analysis stratified by study design, the pooled RRs in case-control and cohort studies were 1.53 (95% CI 1.13–2.06, *I*^2^ = 87.7%, *P*_heterogeneity_ = 0.000) and 0.91 (95% CI 0.68–1.22, *I*^2^ = 36.8%, *P*_heterogeneity_ = 0.209), respectively (Figure 4). In subgroup analysis stratified by continent in which the studies were conducted, the positive association was statistically significant only among studies conducted in North America (RR = 1.21, 95% CI 1.01–1.46, *I*^2^ = 37.8%, *P*_heterogeneity_ = 0.140). In subgroup analysis stratified by dietary assessment method, the positive association was statistically significant in studies that adopted validated FFQs (RR = 1.30, 95% CI 1.02–1.66, *I*^2^ = 85.6%, *P*_heterogeneity_ = 0.000), but not in studies that used FFQs that had not been validated. In the further analysis of NHL subtypes, we just found a statistically significant association between milk consumption and the risk of DLBCL (RR = 1.49, 95% CI 1.08–2.06, *I*^2^ = 8.9%, *P*_heterogeneity_ = 0.333).

**Figure 5:**
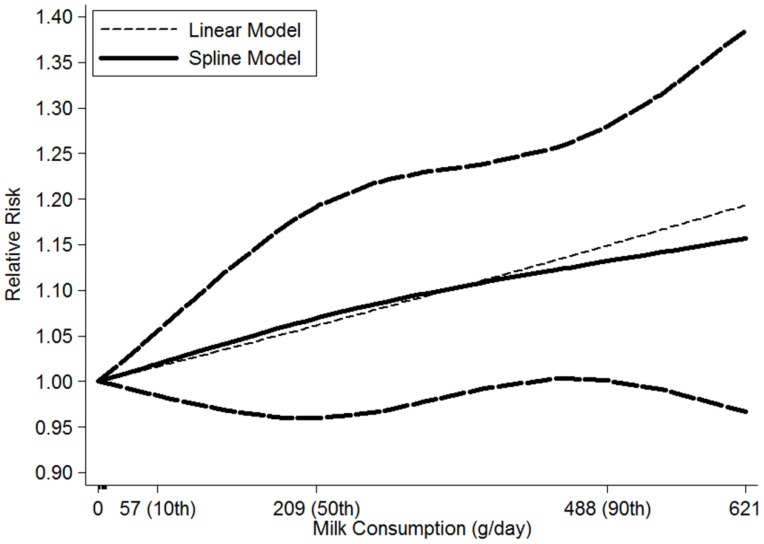
The dose-response analysis between milk consumption and the risk of NHL with restricted cubic splines in a multivariate random-effects dose-response model. The solid line and the long dash line represent the estimated relative risks and its 95% CIs. Short dash line represents the linear relationship. The 10th, 50th and 90th percentiles represent three knots of milk consumption.

For the dose-response analysis, data from nine studies [16,17,18,21,23,27,28] were included, including 3739 NHL cases. A linear relationship was found between milk consumption and the risk of NHL (*P*_nonlinearity_ = 0.78), and the RRs (95% CIs) of NHL were 1.04 (0.97–1.12), 1.07 (0.96–1.19), 1.11 (0.99–1.24), 1.12 (1.00–1.26) and 1.13 (1.00–1.28) for 120, 210, 370, 440 and 490 g/day compared with 0 g/day, respectively. In addition, the dose-response analysis suggested that NHL risk increased by 6% (1.06 (0.99–1.13)) for each 200 g/day increment of milk consumption (Figure 5).

#### 3.2.3. Cheese Consumption and the Risk of NHL

Nine articles [15,16,20,21,23,24,25,26,27] with 10 studies (nine case-control studies and one cohort study) were included, involving 5519 NHL cases. Among these studies, five were conducted in North America, four in Europe and one in Asia. Eight studies adopted validated FFQs to assess cheese consumption and two studies adopted FFQs. The pooled RR of NHL was 1.14 (95% CI 0.96–1.34, *I*^2^ = 58.2%, *P*_heterogeneity_ = 0.011) for the highest *vs.* lowest category of consumption. In subgroup analysis stratified by study design, no association was found in both case-control studies and cohort studies. In subgroup analysis stratified by continent in which the studies were conducted, the positive association was statistically significant only among studies conducted in Europe (RR = 1.28, 95% CI 1.09–1.49, *I*^2^ = 2.2%, *P*_heterogeneity_ = 0.382). In subgroup analysis stratified by dietary assessment method, cheese consumption was associated with an increased risk of NHL (RR = 1.24, 95% CI 1.09–1.40, *I*^2^ = 23.1%, *P*_heterogeneity_ = 0.245) in studies that adopted validated FFQs. In the further analysis of NHL subtypes, we did not find a statistically significant association between cheese consumption and any NHL subtypes.

#### 3.2.4. Other Dairy Product Consumption and the Risk of NHL

For butter consumption, four articles [16,20,21,26] with four case-control studies involving 1534 NHL cases were included, and the pooled RR of NHL was 1.31 (95% CI 1.04–1.65, *I*^2^ = 36.9%, *p*_heterogeneity_ = 0.190) for the highest *vs.* lowest category of consumption. For yogurt consumption, four articles [16,20,21,24] with four studies (three case-control studies and one cohort study) involving 2372 NHL cases were included, and the pooled RR of NHL was 0.78 (95% CI 0.54–1.12, *I*^2^ = 81.6%, *p*_heterogeneity_ = 0.001) for the highest *vs.* lowest category of consumption. In the further analysis of NHL subtypes, we did not find a statistically significant association between yogurt consumption and any NHL subtypes. For ice cream consumption, four articles [16,20,21,28] with four case-control studies involving 1598 NHL cases were included, and the pooled RR of NHL was 1.57 (95% CI 1.11–2.20, *I*^2^ = 72.3%, *P*_heterogeneity_ = 0.013) for the highest *vs.* lowest category of consumption.

### 3.3. Meta-Regression and Influence Analysis

In order to explore the between-study heterogeneity, we performed univariate meta-regression with the covariates of sex, publication year, continent in which the studies were conducted, study design, dietary assessment method and whether the RR (95% CI) was adjusted for energy intake, smoking, alcohol intake and education. In the analysis of total dairy product consumption and the risk of NHL, study design was found to contribute to the between-study heterogeneity (*p* = 0.011). In the analysis of cheese consumption and the risk of NHL, the dietary assessment method was found to contribute to the between-study heterogeneity (*p* = 0.018). None of these covariates was found to have a significant impact on the between-study heterogeneity in other analyses.

In an influence analysis excluding one study at a time, no individual study had an excessive influence on the above-mentioned pooled effects.

### 3.4. Small-Study Effect Evaluation

**Figure 6:**
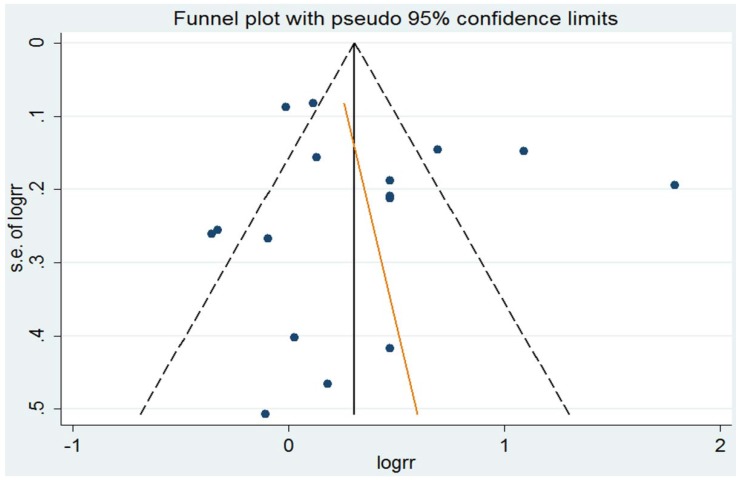
The funnel plot of milk consumption and the risk of NHL. Each dot represents a different study.

Egger’s test showed no evidence of a significant small-study effect for the analyses between the consumption of total dairy product (*p* = 0.402), milk (*p* = 0.616), butter (*p* = 0.798), cheese (*p* = 0.278), yogurt (*p* = 0.196) and ice cream (*p* = 0.250) and the risk of NHL. The funnel plot of the analysis of milk consumption and the risk of NHL was shown in the Figure 6.

## 4. Discussion

This meta-analysis assessed associations between the consumption of total dairy product and specific types of dairy product and the risk of NHL, respectively. Findings from this meta-analysis showed positive associations between the consumption of total dairy product, milk, butter and ice cream and the risk of NHL, respectively. In subgroup analyses that were stratified by study design, only in case-control studies was the consumption of total dairy product and milk associated with an increased risk of NHL. In subgroup analyses that were stratified by the continent in which the studies were conducted, milk consumption was associated with an increased risk of NHL among studies conducted in North America, and cheese consumption was associated with an increased risk of NHL among studies conducted in Europe. When studies were stratified by the dietary assessment method, the consumption of milk and cheese were associated with an increased risk of NHL in those studies that used validated FFQs but not for other dietary assessment methods. Dose-response analysis suggested NHL risk increased by 5% (1.05 (1.00–1.10)) and 6% (1.06 (0.99–1.13)) for each 200 g/day increment of total dairy product and milk consumption, respectively.

The mechanisms underlying the association between dairy product consumption and the risk of NHL have been postulated from several aspects. Dairy product is rich in protein and fat. Some experimental evidence from animal studies suggested that excessive intake of protein could give rise to chronic hyperstimulation of the immune system [39]. Several animal studies have shown that the changes of animal fat and protein in diet can cause impaired immune function [40], which is one of the few well-established risk factors for NHL [41]. An animal study with rats found an increased risk of lymphomas after augmentation of the diet with casein, the major protein of milk [42]. In addition, some epidemiologic studies [43,44] suggested that polychlorinated dibenzo-p-dioxins (PCDDs) and polychlorinated dibenzofurans (PCDFs) could increase the risk of NHL. Several studies indicated that dairy was one of the most important contributors to total PCDDs and PCDFs intake in different regions [45,46,47]. Dairy product is also the main source of calcium. Several studies reported that high calcium intake could restrict the bioavailability of vitamin D [48,49]. An experimental study showed that 1,25-dihydroxyvitamin D_3_, the active form of vitamin D, had an anti-proliferative and pro-differentiation effect on cells that possess vitamin D receptors in follicular lymphoma cell lines [50]. However, a meta-analysis indicated that higher vitamin D did not play a protective role in the risk of NHL [51]. So far, the effect of vitamin D on the association between dairy product consumption and the risk of NHL is still uncertain. More research is needed to confirm this effect. In this meta-analysis, an inverse but not significant association was found between yogurt consumption and the risk of NHL. The anticancer effect of yogurt may depend on the large amounts of lactic acid bacteria, which can enhance the immune response of the host and exert antioxidative and antiproliferative activity [52,53].

Between-study heterogeneity is common in meta-analysis [54]. It is necessary to explore the potential sources of between-study heterogeneity. Our meta-analysis showed different levels of between-study heterogeneities in the analyses of total dairy product and specific types of dairy product consumption and risk of NHL. In meta-regression, we found that study design and the dietary assessment method were the contributors to between-study heterogeneities in the analyses of total dairy product and cheese consumption, respectively. Case-control studies are more susceptible to recall and selection biases than cohort studies. Recall bias is the most common and inevitable bias in all case-control studies, and it refers to the differential recollection of dairy product consumption between cases and controls. The most common selection bias is that the controls are from the hospital. These may contribute to the between-study heterogeneity. Subgroup analysis stratified by dietary assessment method showed that cheese consumption can increase the risk of NHL in studies that adopted validated FFQs. The validated FFQs can provide more accurate dietary intake and make the results more credible. However, meta-regression did not find the sources of between-study heterogeneities in other analyses. The factors accounting for the heterogeneity between studies are complicated. First, the methodologies among the included studies were different, such as the dietary assessment method. Second, the consumption levels ranged widely across the studies included in this meta-analysis. For total dairy product consumption, the lowest intake categories ranged from <3 servings/week to 2.4 servings/day, and the highest intake categories ranged from >1.2 servings/day to 8.5 servings/day. For milk consumption, the lowest intake categories ranged from 0 to <6.9 servings/week, and the highest intake categories ranged from >4 servings/week to >14 servings/week. Third, NHL refers to a heterogeneous group of lymphomas with different prognoses and possible different etiology. The proportion of different NHL subtypes in the included studies may be different. Fourth, the consumption amount and type of dairy product largely differed between Western countries and Eastern countries. All of these factors may contribute to the between-study heterogeneity in concert.

To our knowledge, this is the first meta-analysis to explore the associations between the consumption of total dairy product and specific types of dairy product and the risk of NHL. A major strength of this meta-analysis is the large number of cases included, increasing the statistical power of the study to detect the associations. Second, RRs that reflected the greatest degree of control for potential confounders were extracted, indicating that the results were more credible. Third, considering the potential differences of components in different types of dairy product, we further assessed the effects of specific types of dairy product on the risk of NHL. Fourth, dose-response analysis was conducted to explore the relationships between the consumption of total dairy product and milk and the risk of NHL quantitatively. Fifth, almost all the included studies used a validated FFQ, which ensured the credibility of dietary assessment.

However, there are some limitations in this meta-analysis. First, our results mainly came from case-control studies. There were only three cohort studies included in this meta-analysis, and the positive association between total dairy product consumption and the risk of NHL was not statistically significant in cohort studies. In addition, two other cohort studies [55,56] without RR and/or 95% CI were not included in our meta-analysis. One [55] of them indicated that the consumption of more than two glasses of milk per day could increase the risk of NHL, but another [56] found no statistically significant association between the consumption of any type of dairy product and the risk of NHL. Therefore, more cohort studies with complete data are needed to confirm these results. Second, although major confounders had been adjusted for in most of the included studies, unmeasured and residual confounding was still possible. Confounders adjusted for in each study were also different, which might affect the observed association. Third, the numbers of studies on the consumption of butter, yogurt and ice cream were limited. Thus, fewer cases reduced the statistical power to detect a statistically significant association. Fourth, because of the limited number of studies, we could not evaluate the association between the consumption of dairy product with different fat content and the risk of NHL. Fifth, because of the limited number of studies which examined the associations with specific histopathological subtypes of NHL, we only explored the associations between dairy product consumption and the risk of DLBCL, follicular lymphoma (FL), and small lymphocytic lymphoma/chronic lymphocytic leukemia (SLL/CLL). The fewer studies and cases reduced the statistical power to detect statistically significant associations. Sixth, in dose-response analysis, we assumed that the amplitude of the highest category is same as the contiguous category. This may be imprecise given the asymmetric distributions of food consumption. Considering that most of the studies in dose-response analysis were conducted in the US, we adopted standard units reported in the United States Department of Agriculture National Nutrient Database for Standard Reference to convert our data for all the included studies. These may affect the observed association to some extent.

## 5. Conclusions

In summary, this meta-analysis suggested that dairy product, but not yogurt, may increase the risk of NHL. The risk of NHL increased by 5% and 6% for each 200 g/day increment of total dairy product and milk consumption, respectively. The results mainly came from case-control studies, and thus more cohort studies focusing on specific types of dairy product consumption are needed to confirm the conclusion.

## Figures and Tables

**Table 1:** Characteristics of case-control studies on dairy product consumption and the risk of NHL.

Author	Country (Year)	Age	Dietary Assessment	Participants (Cases)	Gender	Exposure	Outcome	RR (95% CI)	Adjustment for Covarianes
Balasubram-aniam, G. [14]	India (2013)	Cases 46.1 Controls 46.4 (mean)	FFQ	1726 (348)	M	Milk	NHL	6.00 (4.10, 8.80)	Cigarette smoking, bidi smoking, tobacco lime chewing, the consumption of coffee, chicken, red-meat, eggs, fish, chilly and vegetable, and exposure to pesticides and cotton dust
Zheng, T. [28]	America (2004)	21–84	Validated FFQ	1318 (601)	W	Total dairy	NHL	1.80 (1.20, 2.80)	Age, BMI, family history of NHL in first-degree relatives, and total energy intake
				1305 (594)		Milk	NHL	1.60 (1.00, 2.30)	
				1205 (494)		Ice cream	NHL	1.50 (1.10, 2.10)	
Tavani, A. [26]	Italy (1997)	Cases 58 Controls 57 (median)	Validated FFQ	1586 (429)	M&W	Milk	NHL	2.00 (1.50, 2.66)	None
						Cheese	NHL	1.37 (1.03, 1.82)	
						Butter	NHL	1.78 (1.21, 2.62)	
Ali, A. [13]	Oman (2013)	NA	Validated FFQ	86 (43)	M&W	Total dairy	NHL	0.81 (0.29, 2.23)	Age and sex
Ward, M.H. [27]	America (1994)	≥21	FFQ	714 (171)	M	Milk	NHL	1.60 (0.70, 3.60)	Age
						Cheese	NHL	0.60 (0.40, 1.00)	
				676 (144)	W	Milk	NHL	1.20 (0.50, 3.10)	
						Cheese	NHL	0.70 (0.40, 1.30)	
Chang, E.T. [15]	Sweden (2005)	Cases 62 Controls 59 (median)	Validated FFQ	1064 (597)	M&W	Total dairy	NHL	1.50 (1.10, 2.20)	Age and sex
				595 (128)			DLBCL	2.00 (1.20, 3.50)	
				572 (105)			FL	1.20 (0.60, 2.20)	
				614 (147)			SLL/CLL	1.50 (0.90, 2.60)	
				1064 (597)		Milk	NHL	1.60 (1.10, 2.50)	
				1064 (597)		Cheese	NHL	1.40 (1.00, 2.00)	
Purdue, M.P. [23]	Canada (2004)	20–74	Validated FFQ	5616 (1631)	M&W	Milk	NHL	1.12 (0.95, 1.31)	Age, sex, income adequacy, alcohol consumption, and total energy
				5554 (1616)		Cheese	NHL	1.38 (1.06, 1.53)	
Talamini, R. [25]	Italy (2006)	Cases 58 Controls 63 (median)	Validated FFQ	674 (190)	M&W	Milk Cheese	NHL NHL	0.91 (0.54, 1.54) 1.66 (0.98, 2.83)	Age, sex, center, education, place of birth, hepatitis C virus test, and total energy intake
De Stefani, E. [19]	Uruguay (2013)	NA	Validated FFQ	3975 (369)	M&W	Milk	NHL	2.98 (2.23, 3.98)	Age, sex, residence, urban/rural status, education, BMI, smoking intensity, total meat, alcohol drinking, mate consumption, total meat, and total energy
Ollberding, N.J. [21]	America (2013)	Cases 58.6 Controls 58 (mean)	Validated FFQ	793 (333)	M&W	Total dairy	NHL	1.50 (1.10, 2.20)	Age, sex, educational attainment and total energy
				548 (88)			DLBCL	1.40 (0.80, 2.60)	
				564 (104)			FL	1.50 (0.90, 2.60)	
				485 (25)			SLL/CLL	3.00 (0.90, 9.50)	
				793 (333)		Milk	NHL	1.60 (1.10, 2.30)	
				548 (88)			DLBCL	1.80 (1.00, 3.10)	
				564 (104)			FL	1.90 (1.10, 3.20)	
				485 (25)			SLL/CLL	2.30 (0.90, 6.00)	
				793 (333)		Cheese	NHL	0.90 (0.70, 1.30)	
				548 (88)			DLBCL	0.90 (0.50, 1.60)	
				564 (104)			FL	1.00 (0.60, 1.70)	
				485 (25)			SLL/CLL	1.50 (0.50, 4.20)	
				793 (333)		Ice cream	NHL	1.40 (1.00, 2.00)	
				793 (333)		Yogurt	NHL	0.80 (0.50, 1.20)	
				548 (88)			DLBCL	0.60 (0.30, 1.20)	
				564 (104)			FL	0.60 (0.40, 1.20)	
				485 (25)			SLL/CLL	1.60 (0.50, 5.60)	
				793 (333)		Butter	NHL	1.00 (0.70, 1.40)	
De Stefani, E. [18]	Uruguay (1998)	20–84	FFQ	171 (85)	M	Milk	NHL	1.03 (0.47, 2.28)	Age, residence, urban/rural status, type of tobacco, beer intake and “mate“/years Age, residence, urban/rural status, year of diagnosis and parity
				152 (75)	W		NHL	0.90 (0.33, 2.41)	
Charbonneau, B. [16]	America (2013)	Cases 60.9 Controls 60.1 (mean)	Validated FFQ	1609 (602)	M&W	Total dairy	NHL	1.12 (0.79, 1.60)	Age, sex, residence, and total energy
				1112 (105)			DLBCL	1.83 (0.89, 3.75)	
				1153 (146)			FL	0.98 (0.55, 1.76)	
				1225 (218)			SLL/CLL	0.88 (0.52, 1.47)	
				1609 (602)		Milk	NHL	1.14 (0.84, 1.55)	
				1112 (105)			DLBCL	1.85 (1.01, 3.40)	
				1153 (146)			FL	0.99 (0.58, 1.70)	
				1225 (218)			SLL/CLL	0.84 (0.54, 1.32)	
				1609 (602)		Cheese	NHL	1.12 (0.81, 1.57)	
				1112 (105)			DLBCL	1.06 (0.52, 2.12)	
				1153 (146)			FL	0.94 (0.53, 1.69)	
				1225 (218)			SLL/CLL	1.18 (0.73, 1.91)	
				1609 (602)		Ice cream	NHL	2.45 (1.80, 3.34)	
				1609 (602)		Yogurt	NHL	1.01(0.77, 1.33)	
				1112 (105)			DLBCL	0.88 (0.49, 1.57)	
				1153 (146)			FL	1.12 (0.70, 1.81)	
				1225 (218)			SLL/CLL	0.99 (0.68, 1.45)	
				1609 (602)		Butter	NHL	1.29 (0.99, 1.69)	
Mozaheb, Z. [20]	Iran (2012)	Cases 51 Controls 47 (mean)	Validated FFQ	360 (170)	M&W	Milk	NHL	0.72 (0.44, 1.20)	None
						Cheese	NHL	1.38 (0.79, 2.40)	
						Ice cream	NHL	1.05 (0.65, 1.71)	
						Yogurt	NHL	0.32 (0.18, 0.55)	
						Butter	NHL	1.34 (0.76, 2.37)	

Abbreviations: RR, relative risk; CI, confidence interval; BMI, body mass index; FFQ, food frequency questionnaire; M, men; W, women; NHL, non-Hodgkin lymphoma; DLBCL, diffuse large B-cell lymphoma; FL, follicular lymphoma; SLL/CLL, small lymphocytic lymphoma/chronic lymphocytic leukemia; NA, not available.

**Table 2:** Characteristics of cohort studies on dairy product consumption and the risk of NHL.

Author	Country (Year)	Age	Dietary Assessment	Participants (Cases)	Gender	Exposure	Outcome	RR (95% CI)	Adjustment for Covarianes
Rohrmann, S. [24]	10 European countries (2011)	M 52.7 W 50.8 (median)	Validated FFQ	410,411 (1267)	M&W	Milk	NHL	0.99 (0.83, 1.17)	Energy, alcohol, education, fruits, vegetables and smoking
				410,411 (159)			DLBCL	1.14 (0.72, 1.82)	
				410,411 (140)			FL	0.51 (0.29, 0.90)	
				410,411 (234)			SLL/CLL	0.96 (0.66, 1.42)	
				410,411 (1267)		Cheese	NHL	1.09 (0.86, 1.40)	
				410,411 (159)			DLBCL	0.85 (0.40, 1.83)	
				410,411 (140)			FL	1.28 (0.65, 2.50)	
				410,411 (234)			SLL/CLL	1.38 (0.79, 2.42)	
				410,411 (1267)		Yogurt	NHL	1.02 (0.88, 1.17)	
				410,411 (159)			DLBCL	1.04 (0.70, 1.56)	
				410,411 (140)			FL	0.97 (0.63, 1.48)	
				410,411 (234)			SLL/CLL	0.92 (0.67, 1.28)	
Chiu, B.C. [17]	America (1996)	55–69	Validated FFQ	35,156 (104)	W	Total dairy	NHL	1.04 (0.61, 1.77)	Age and total energy intake
						Milk	NHL	0.70 (0.42, 1.17)	
Park, Y. [22]	America (2009)	50–71	Validated FFQ	293,907 (1267)	M	Total dairy	NHL	1.02 (0.85, 1.22)	Race/ethnicity, education, marital status, BMI, family history of cancer, vigorous physical activity, alcohol consumption, intakes of red meat and total energy, and smoking
				198,903 (660)	W		NHL	1.00 (0.77, 1.30)	

Abbreviations: RR, relative risk; CI, confidence interval; BMI, body mass index; FFQ, food frequency questionnaire; M, men; W, women; NHL, non-Hodgkin lymphoma; DLBCL, diffuse large B-cell lymphoma; FL, follicular lymphoma; SLL/CLL, small lymphocytic lymphoma/chronic lymphocytic leukemia.3.2. Quantitative Synthesis

**Table 3:** Summary risk estimates of the association between dairy product consumption and the risk of NHL and NHL subtypes.

Exposure	Outcome	Subgroup	No. of Studies	No. of Cases	Pooled RR (95% CI)	*I*^2^ (%)	*p*_heterogeneity_
Total dairy product							
Total dairy product	NHL	All studies	8	4207	1.20 (1.02, 1.42)	42.7	0.094
		Study design					
		Case-control	5	2176	1.41 (1.17, 1.70)	6.8	0.368
		Cohort	3	2031	1.02 (0.88, 1.17)	0.0	0.988
		Continent					
		North America	6	3567	1.17 (0.98, 1.40)	46.6	0.095
		Europe	1	597	1.50 (1.06, 2.12)	NA	NA
		Asia	1	43	0.81 (0.29, 2.25)	NA	NA
	DLBCL	All studies	3	321	1.73 (1.22, 2.45)	0.0	0.670
	FL	All studies	3	355	1.23 (0.88, 1.72)	0.0	0.569
	SLL/CLL	All studies	3	390	1.35 (0.77, 2.39)	53.8	0.115
Specific type of dairy product							
Milk	NHL	All studies	16	7109	1.41 (1.08, 1.84)	88.6	0.000
		Study design					
		Case-control	14	5738	1.53 (1.13, 2.06)	87.7	0.000
		Cohort	2	1371	0.91 (0.68, 1.22)	36.8	0.209
		Continent					
		North America	7	3579	1.21 (1.01, 1.46)	37.8	0.140
		Latin America	3	529	1.53 (0.63, 3.70)	80.5	0.006
		Europe	4	2483	1.32 (0.87, 1.98)	85.0	0.000
		Asia	2	518	2.09 (0.26, 16.71)	97.7	0.000
		Dietary assessment					
		Validated FFQ	11	6286	1.30 (1.02, 1.66)	85.6	0.000
		FFQ	5	823	1.68 (0.67, 4.20)	87.3	0.000
	DLBCL	All studies	3	352	1.49 (1.08, 2.06)	8.9	0.333
	FL	All studies	3	390	0.99 (0.47, 2.07)	81.8	0.004
	SLL/CLL	All studies	3	477	1.04 (0.69, 1.55)	44.1	0.167
Cheese	NHL	All studies	10	5519	1.14 (0.96, 1.34)	58.2	0.011
		Study design					
		Case-control	9	4252	1.14 (0.94, 1.38)	61.9	0.007
		Cohort	1	1267	1.09 (0.85, 1.39)	NA	NA
		Continent					
		North America	5	2866	0.95 (0.70, 1.29)	75.7	0.002
		Europe	4	2483	1.28 (1.09, 1.49)	2.2	0.382
		Asia	1	170	1.38 (0.79, 2.41)	NA	NA
		Dietary assessment					
		Validated FFQ	8	5204	1.24 (1.09, 1.40)	23.1	0.245
		FFQ	2	315	0.64 (0.44, 0.91)	0.0	0.686
	DLBCL	All studies	3	352	0.93 (0.63, 1.37)	0.0	0.905
	FL	All studies	3	390	1.04 (0.74, 1.46)	0.0	0.777
	SLL/CLL	All studies	3	477	1.28 (0.91, 1.81)	0.0	0.876
Butter	NHL	All studies	4	1534	1.31 (1.04, 1.65)	36.9	0.190
Yogurt	NHL	All studies	4	2372	0.78 (0.54, 1.12)	81.6	0.001
	DLBCL	All studies	3	352	0.90 (0.67, 1.21)	0.0	0.402
	FL	All studies	3	390	0.89 (0.63, 1.25)	33.9	0.220
	SLL/CLL	All studies	3	477	0.97 (0.76, 1.23)	0.0	0.679
Ice cream	NHL	All studies	4	1598	1.57 (1.11, 2.20)	72.3	0.013

Abbreviations: RR, relative risk; CI, confidence interval; FFQ, food frequency questionnaire; NHL, non-Hodgkin lymphoma; DLBCL, diffuse large B-cell lymphoma; FL, follicular lymphoma; SLL/CLL, small lymphocytic lymphoma/chronic lymphocytic leukemia; NA, not available.
